# When Food Co-Branding Backfires: The Overexpectation Effect

**DOI:** 10.3390/foods11142136

**Published:** 2022-07-19

**Authors:** Marion Garaus, Elisabeth Wolfsteiner, Arnd Florack

**Affiliations:** 1 Department of International Management, Modul University Vienna, Am Kahlenberg 1, 1190 Vienna, Austria; marion.garaus@modul.ac.at; 2 Department of Innovation, University of Applied Sciences Wiener Neustadt, Zeiselgraben 4, 3250 Wieselburg, Austria; elisabeth.wolfsteiner@fhwn.ac.at; 3 Department of Occupational, Economic, and Social Psychology, University of Vienna, Universitaetsstraße 7, 1010 Vienna, Austria

**Keywords:** co-branding, brand evaluation, brand names, brand associative learning

## Abstract

While food research has paid considerable attention to the effect of brand names on brand evaluation, the role of co-branding strategies and hence simultaneous exposure to two different brand names is under-researched. Against this background, we investigated the overexpectation effect in the context of food co-branding. More specifically, we explored to what extent food co-branding can harm brand evaluations of the co-brand and the brand level of the partner. In doing so, we challenged the conventional wisdom that co-branding leads to higher brand evaluations than those of monobrands. Results from two online experiments confirmed the theoretical reasoning derived from adaptive learning models: combining two brands results in an overexpectation effect, which manifests in a decrease in levels of brand evaluation for the co-brand compared to the partnering brands before co-brand exposure. Brand strength and brand fit moderate this effect.

## 1. Introduction

Introducing new food and beverage products is associated with a high risk of failure. Indeed, research shows the failure rate of introductions in the food and beverage sector is between 50–75%, with considerable loss of time and money [1]. The introduction of new products is impeded by these high failure rates as well as high levels of competition from strong and established brands. For instance, 60% of the world’s chocolate market is owned by only six brands [2]. Nevertheless, in 2019, 20,062 new food and beverage products were introduced globally [3]. This competitive environment requires the food industry to add value by the creation of brands [4]. The proliferation of products in the food and beverage sector hampers the introduction of new products and makes the process of choosing between them more difficult for consumers [5]. In situations with a wide variety of choices, familiar brand names decrease the difficulty of making a decision [6]. External cues, such as brand names or brand labels, represent an important predictor of purchasing decisions [7,8] and favorable brand evaluations [9,10]. Brand names are often used to infer a product’s quality prior to consumption [11].

Given the relevance of brand names for predicting consumers’ perceptions of quality and brand evaluations, co-branding has become an omnipresent strategy to use the benefits of one brand for the benefits of another. Co-branding describes the use of two or more brand names on one product [12]. Evidence shows that many brands of moderate strength rely on a co-branding strategy to benefit from the awareness of and associations with a strong monobrand [13]. Monobrands refer to the partnering brands of the co-branding arrangement. As well as cost-savings and access to the other brand’s market [14], studies identify increased perceptions of the quality of the co-branded product (e.g., [15]) and a rise in brand equity (e.g., [16,17]) as the major advantages of co-branding. Accordingly, common wisdom suggests that a co-branded product would be evaluated as highly as the average evaluation of each of the two brands, or even higher. A co-brand is assumed to bundle together the benefits of each brand, causing an increase in brand evaluation. Hence, co-branding is assumed to be a promising strategy to increase brand evaluation. While this assumption is reasonable, we suggest a contrasting perspective: the combination of two brands with strong benefits might cause a decrease in brand evaluations. This phenomenon has been discussed theoretically under the terminology overexpectation [18]. The overexpectation effect is based on adaptive learning models, which suggest that the interaction of cues can prompt learning processes that intensify or weaken the strength between the cues and a specific outcome [19]. 

In a co-branding context, the inflated expectation of a doubled outcome (i.e., benefits from both brands) cannot be met by the co-brand and results in a discrepancy between the expected and the actual benefits. In such a situation, the benefits of the brand it is being partnered with need to be adjusted downwards, so that this discrepancy on a co-brand level is reduced [20,21]. By way of illustration, a co-brand of a famous yogurt brand and a famous chocolate brand might cause consumers to expect an entirely new taste experience. However, when exposed to the co-brand, it might become obvious that the co-branded product does not differ as expected from the original yogurt (without the famous chocolate brand). In other words, the co-brand is evaluated as being worse than the average of the two partnering monobrands. Extant evidence reports that co-branding does not result in the expected outcomes, but shows a lower willingness to pay [22,23], lower perceptions of quality of subsequent co-branding between the partnering brands [15], or the absence of any effect on brand evaluations [24]. However, only a few studies refer to adaptive learning models to explain the potential negative feedback effects of co-branding strategies [13,15]. Indeed, most studies explore co-branding from the common wisdom perspective, i.e., that merging two brands increases brand evaluations. Negative effects are often observed incidentally, and theoretical explanations for these negative effects are missing. Accordingly, there is limited evidence on the applicability of adaptive learning models in the field of co-branding. Nevertheless, a better understanding of the underlying processes that prompt negative co-branding outcomes would not only enhance knowledge of consumer learning processes but would also help brand managers to optimize their strategic branding decisions. Hence, one major objective of the current research is to shed light on the underlying mechanism which causes lower co-brand evaluations compared to the monobrand’s evaluation before the co-branding. 

The negative effect of co-branding strategies might not occur for all brands to the same extent. Indeed, extant literature is inconclusive on the positive and negative effects of co-branding strategies on brand evaluation. Hence, the identification of boundary conditions on potential negative effects of co-branding would further enhance the knowledge of the circumstances that cause the overexpectation effect.

The extant literature identifies brand strength and brand fit as relevant for co-branding success [12,15,25,26,27]. Following this research, we consider both variables (brand strength and brand fit) as potential moderators determining co-branding success. Strong brands are associated with favorable benefits, which makes an overexpectation effect caused by co-branding strategies even more likely. Strong benefits from the partnering brands result in a higher discrepancy between expected and actual benefits compared to moderate or weak benefits. Accordingly, the benefits of the partnering brands require more downward adjustment for stronger brands compared to weaker brands [15]. Thus, another important aim of the current research is to investigate if negative effects occur for strong and moderate brands alike. The differentiation between strong and moderate brands allows the testing of the theoretical reasoning of this study that the overexpectation is pronounced for strong brands and hence would offer further empirical evidence on the applicability of adaptive learning models in a co-branding context. 

Brand fit determines co-branding success, as higher fit levels result in more favorable outcomes [17,28]. One way to operationalize brand fit is by assessing brand complementarity [29]. In line with this suggestion, we consider the level of complementarity as brand fit, which refers to “consumers’ perception of the necessity of one product for the performance or use of the second product” [30] (p. 59). Park, Jun, and Shocker [26] reveal that two strong partnering brands can increase their attribute profile when paired with each other only when the two brands are complementary. Other research confirms the positive effect of attribute complementarity in co-branding [30,31]. We propose that low fit levels imply that the partnering brand’s benefits are low in terms of complementarity, which fosters the overexpectation effect. In other words, if both brands are strong owing to the same attribute, consumers might have unrealistic expectations of this attribute for the co-brand. On the contrary, if the two partnering brands are known for different attributes, the overexpectation effect is attenuated since it will not result in a doubling up of one particular brand attribute on the co-brand level; instead it will add a new positive attribute to an already-existing one. A new product attribute can develop a new association between the brand and the outcome while promoting an existing attribute will exceed its predictive value.

Co-branding strategies do not only strive for a favorably evaluated co-brand. Often, co-branding efforts seek to generate long-lasting effects for the partnering monobrand (i.e., spillover effects). Nevertheless, not all co-branding arrangements result in favorable long-term effects. Some studies report detrimental effects for the monobrands after co-brand exposure; for example, respondents wanted to pay lower amounts of money for the header brand after exposure to the co-branded product [23]. Likewise, if a monobrand has already been experienced as part of a co-branded product, another co-branding with this brand does not benefit from it anymore [15]. In the context of brand extensions, such a negative evaluation has become well-known as a negative feedback effect [32]. In the current context, a negative feedback effect implies that the negative consumer evaluations of the co-brand spill over to the monobrand [33]. If co-branding strategies fail, negative feedback effects occur at the monobrand level, with this effect being stronger for moderately strong monobrands [34]. Against this background, the overall objective of the current research is to shed light on the underlying mechanism which causes lower co-brand and monobrand evaluations compared to the monobrand’s evaluation before the co-branding, and to investigate the moderating role of brand fit and brand strength. More formally, we propose the following hypotheses:

**Hypothesis** **1.** A co-brand is evaluated as being worse than the average of the two partnering brands.

**Hypothesis** **2.** Brand fit moderates this effect, so that high levels of brand fit attenuate the negative effect.

**Hypothesis** **3.** Brand strength moderates this effect, so that co-brands consisting of moderately strong monobrands attenuate the negative effect.

**Hypothesis** **4.** A monobrand is evaluated as being worse as after the exposure of individuals to the co-brand.

**Figure 1:**
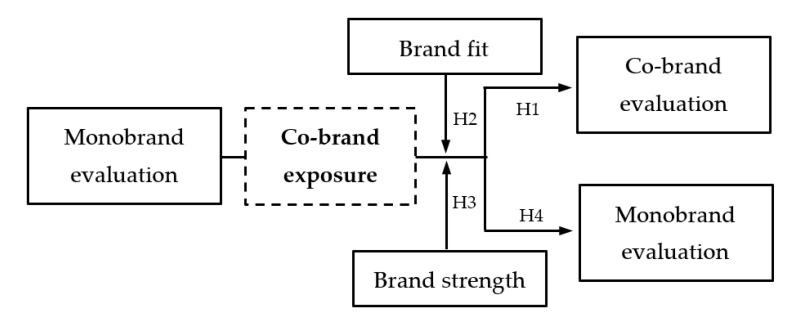
Conceptual research framework.

Figure 1 illustrates the conceptual research framework. 

## 2. Materials and Methods

The hypotheses were tested with two sets of panel data (representative of the population under investigation) gained by two online experiments with varying brands from six different product categories as stimulus materials. More specifically, experiment 1 tested H1, H2, and H4 using a pastry brand and a chocolate brand as a stimuli. Experiment 2 tested H1, H2, H3, and H4 with 12 different brands as stimuli from the product categories pastry, juices, mineral water, beer, chocolate, and soft drinks. For each product category, one popular and one less popular brand were selected based on the Brand Equity Index calculated by the Nielsen Market Track and as reported in the Cash magazine [35]. To further validate the popularity of the strong and moderate brands, more current data from Statista were used to complement Nielsen’s Brand Equity Index (see Table 1). Brand quality and brand evaluation were measured as dependent variables and summarized as a composite score for the main analysis. 

### 2.1. Experiment 1

Study 1 investigates the basic assumption that a co-brand is evaluated as being worse than its monobrands after being presented to individuals (H1). Another aim of study 1 is to test the moderating effect of brand fit (H2) and to test the effect of co-brand exposure on the monobrands (H4). We subsequently presented respondents with monobrands and co-brands, which were promoted with positive product attributes in an advertisement to create a realistic situation of brand exposure. To further enhance the generalizability of our results, we used two brands familiar to the population under investigation. We also varied the positive attribute used to promote the co-brand, so that we either used an attribute from one or the other monobrand. To avoid any sequence effects, the order of presentation of the two monobrands was varied as well. We measured brand evaluation, i.e., monobrand vs. co-brand, as an outcome of our experimental manipulation. 

#### 2.1.1. Participants and Design 

The study employed a one-factor within-subjects design, with time of measurement representing the factor variable. A representative sample of 195 Austrian consumers (stratified according to age, gender, and education) was collected through an online panel (Talk Online panel). The minimum number of participants (*p* = 0.05, effect size: 0.25) required was determined as being 43 respondents by an a priori power analysis (Gpower: [42]). Members of the online panel were invited to take part in a survey exploring the evaluation of different food brands. The survey took, on average, six minutes. Of the participants, 47.7% identified as female and the average age was 43. Participants had different educational backgrounds; 8.7% had a university degree, 19% had graduated from high school, 26.2% had completed vocational schooling, 39.5% had completed an apprenticeship, and 6.7% had finished compulsory schooling. Participants were assigned randomly to the different variations in terms of attribute promotion and sequence of presentation of the monobrand.

#### 2.1.2. Materials and Procedure 

Two brands selling chocolate and pastry products (Milka and Ölz) were selected as stimuli. For Milka, we promoted the claim “unforgettably tender” and for Ölz, we promoted the claim “made with love” on the advertisement together with the brand logo. We further varied the color of the advertisement and the slogan to fit either Milka or Ölz. 

**Figure 2:**
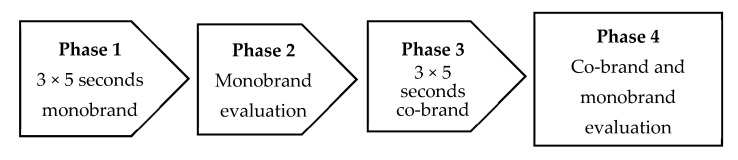
Experimental design of study 1.

The experimental procedure consisted of four phases (see Figure 2). In the first phase, we presented the monobrand together with the corresponding brand claim three times in the form of a (slightly adapted) real-world advertisement for five seconds. Afterwards, in Phase 2, participants evaluated the presented monobrands. Between the presentations, participants were asked to complete a distraction task (an anagram). In phase 3, participants were exposed three times for five seconds to the co-brand promoted with a brand attribute, followed by another evaluation phase (phase 4) where participants evaluated the co-brand and the monobrand based on the same two items as in phase 2. 

#### 2.1.3. Measures

We measured brand evaluation using the following two items: “*How do you generally evaluate this branded product?*” (−4 negative to +4 positive) and “*How do you evaluate the quality of the brand?*” (−4 very low to +4 very high, [13,15]). We collapsed the evaluation of the two monobrands to a single overall evaluation score (Cronbach’s *α* = 0.89). Finally, we assessed brand fit, measured with the question: “*How well do the monobrands of this co-branded product fit together?*” [43]. 

#### 2.1.4. Data Analysis 

Our main hypothesis was that the co-brand would receive worse evaluations than the monobrand. To test this hypothesis (H1), we used the collapsed overall brand evaluation score. We then estimated an RM ANCOVA. The RM ANCOVA included brand evaluation as the dependent variable and the three measurement points as a factor variable. Furthermore, we assessed the moderating role of brand fit by considering this variable as covariate. Furthermore, we assessed the postulated moderating effect of brand fit (H2) by a moderation analysis (model 2) using the MEMORE macro from Montoya [44] to compare the monobrand evaluation in phase 2 and the co-brand evaluation in phase 4. Another objective of study 1 was to test H4, which postulated the monobrand as being evaluated as worse after the exposure of individuals to the co-brand (i.e., monobrand evaluation in phase 2 vs. phase 4). This hypothesis was tested by another moderation analysis (MEMORE macro, model 2). 

#### 2.1.5. Results

**Figure 3:**
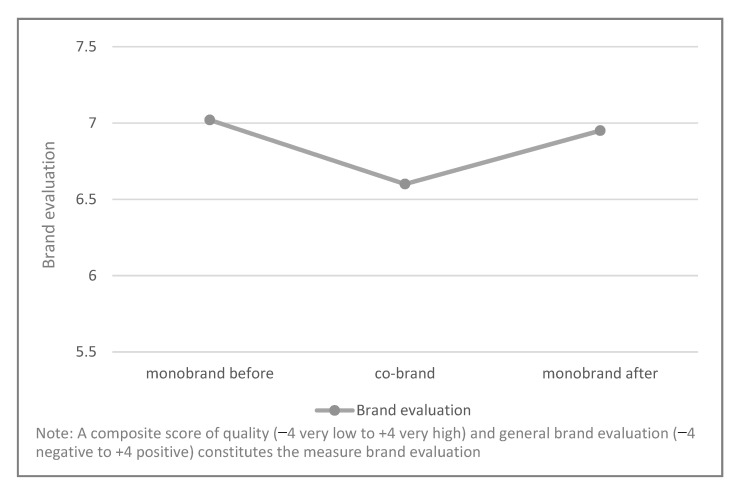
Mean comparisons of study 1.

Supporting H1, the analysis revealed a significant effect of point of measurement on brand evaluations (Pillai’s trace = 0.37, *F*(2, 192) = 56.46, *p* < 0.01, *η_p_*^2^ = 37). This finding is validated by paired-sample t-tests: The co-branded products are evaluated as significantly worse than the average of the strong monobrands before the co-branding strategy (*M_mon_obefore_* = 7.02, *SD* = 1.53 vs. *M_co-brand_* = 6.60, *SD* = 1.90, *p* < 0.01). This result corroborates H1 (see Figure 3). 

This effect was moderated by brand fit as revealed by a significant interaction between time of measurement and brand fit (Pillai’s trace = 0.30, *F*(2, 192) = 41.42, *p* < 0.01). The moderation analysis (MEMORE macro, model 2) comparing the monobrand evaluation in phase 2 and the co-brand evaluation in phase 4 revealed that this effect is moderated by brand fit (−0.27, CI[−0.34, −0.21); the negative effect of co-branding is pronounced if the two monobrands do not fit with each other (*F*(1, 193) = 73.19, *p* < 0.01, *R*^2^ = 0.28). For each unit increase in fit, there is a 0.27 decrease in the difference between the previous evaluation of the monobrand before and the co-brand. An inspection of the conditional effects of the value of the moderator reveals that the difference between the monobrand and the co-brand evaluation is pronounced at low and moderate levels of fit (*β_low_fit_* = 0.96, *p* < 0.01 CI [0.78, 1.14]; *β_moderate_fit_* = 0.42, *p* < 0.01, CI [0.29, 0.54]), while no significant moderating effect was observed for high levels of fit (*β_highfit_* = −0.13, *p* = 0.16, CI [−0.30, 0.05]). This result collaborates H2. 

Testing H4, the analysis reveals that the average of the two partnering monobrands received almost the same evaluation as before (*M_mono_before_* = 7.03, *SD* = 1.53 vs. *M_mono_after_* = 6.95, *SD* = 1.65, *p* = 0.19, *η_p_*^2^ = 0.09). A further moderation analysis (model 2) using the MEMORE macro revealed that the evaluation of the monobrand in phase 4 remains stable when accounting for a potential moderating effect of brand fit (−0.02, CI [−0.07, 0.02]). Accordingly, H4 is not supported (see Figure 3).

### 2.2. Experiment 2

Study 2 sought to validate the results of study 1. In addition, another objective of study 2 was to investigate the moderating effect of brand strength (H3). In a similar fashion to experiment 1, we presented participants with food brands; however, in contrast to the first study, we used twelve different brands to enhance the generalizability of our results. Each respondent was exposed to a set of six brands, and two of these six monobrands were used to develop a fictitious co-brand. Importantly, we also manipulated the brand strength of the monobrands and co-brands by using monobrands with varying brand equity. 

#### 2.2.1. Participants and Design

The study employed a two-factor within-subjects design, with time of measurement representing one factor variable and brand strength representing the other factor variable. A power analysis [45] confirmed that a minimum sample size of 103 would be necessary to achieve 80% power (*α* = 0.05). A sample of 131 online panelists (mean age = 43, 62 of whom identified as female) was acquired through an online panelist provider (Talk Online Panel). The sample represented the population of the country under investigation in terms of age and gender. A power analysis confirmed that the sample size was sufficient to detect effects of 0.25 (*p* = 0.05). The survey’s content was announced with the evaluation of various brands. The educational backgrounds varied among the respondents; the majority had completed an apprenticeship (35.1%), 28.2% had completed vocational schooling, 19.1% indicated high school as their highest level of education, 10.7% had graduated from a university, and 6.9% had finished compulsory schooling. Participants were randomly allocated to the two different sets of strong and moderate brands.

#### 2.2.2. Materials and Procedure 

Pictures of twelve real-world food products were selected as stimuli (see Table 1). For instance, in the flavored mineral water category, Vöslauer (mineral water) and Rauch (juice) presented strong brands and therefore built a strong co-brand. In contrast, combining Rogaska (mineral water) with Mein Obst (juice; two moderate brands) reflected a moderate co-brand in the category juice. 

**Figure 4:**
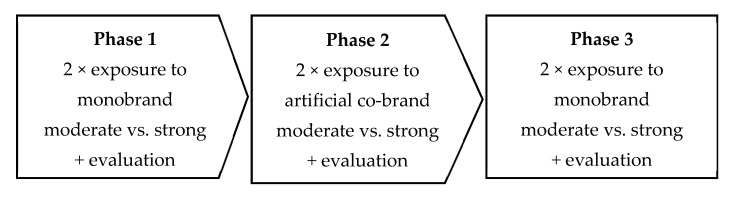
Experimental design of study 2.

The experimental setup was similar to study 1, with three exceptions (see Figure 4). First, pictures of real-world food products served as a stimulus instead of presenting the brand in the form of an advertisement. Second, respondents were exposed to either two strong and four moderate brands or four strong and two moderate brands in the categories of juice, alcoholic drinks, snacks, and pastries in phase 1, and the evaluation of the brand followed immediately after the presentation of the brand. More specifically, respondents were shown each brand two times. After the first time, an open-text field asked for the brand name as an attention check. Next, the monobrand was presented again, and on the same page, participants evaluated the brand. As a third difference, no distraction task followed exposure to the brand. Afterwards, in phase 2, three artificial co-brands were given. The co-brands were created by adding the logo of one monobrand to the package of the other monobrand. For instance, the brand Rauch was placed on the label of the mineral water Rogaska to simulate a co-branded flavored mineral water constituting two monobrands (Rauch and Rogaska). Finally, in phase three, participants were shown the same set of monobrands followed by the same measurement items from phase 1.

#### 2.2.3. Measures

We measured brand evaluation using the same questions as study one: two items assessing brand evaluation, “*How do you generally evaluate this branded product?*” (−4 negative to +4 positive) and “*How do you evaluate the quality of the brand?*” (−4 very low to +4 very high, [13,15]). A satisfying reliability analysis (Cronbach’s *α* = 0.93) justified collapsing the evaluation of the monobrands. The single item “*How well do the monobrands of this co-branded product fit together*” measured brand fit [43]. Finally, another single item assessed the frequency of prior purchases of this product *“How often have you purchased this product in the last 6 months?”*

#### 2.2.4. Data Analysis 

The brand evaluations are nested within participants due to the study design (i.e., participants evaluated multiple food brands). To account for these issues, we estimated a mixed linear-effect model in SPSS with a significance level of α = 0.05 to test our hypotheses. We specified brand evaluation as a dependent variable and brand strength (strong vs. moderate) and time of measurement (before co-brand exposure, co-brand, after co-brand exposure) as factor variables. We also controlled for the frequency of previous purchases of the product and fit between the two monobrands that constitute the co-brand. Participants served as random effects. We further estimated two moderation models to assess the postulated moderating role of brand fit on the two measurement points: monobrands before exposure to the co-brand (phase 1) and the co-brand evaluation (phase 2), and on the two measurements points: moderate monobrand before (phase 1) and moderate monobrand after (phase 4).

#### 2.2.5. Results

In support of H1, the model revealed a main effect of time ((before co-brand exposure, co-brand, after co-brand exposure), (*F*(2, 1572) = 33.57, *p* < 0.01). Furthermore, the analysis revealed a main effect of strength, (*F*(1, 786) = 45.85, *p* < 0.01, and a significant interaction effect of brand strength × time, (*F*(2, 1572) = 8.50, *p* < 0.01), confirming H3. More specifically, the analysis revealed that only strong co-brands were evaluated as being worse than the average of the two strong monobrands, while the evaluation of moderate co-brands did not differ significantly from the average of the two moderate monobrands. 

Both covariates, brand fit (*F*(1, 786) = 301.97, *p* < 0.01) and frequency of prior purchase (*F*(1, 786) = 39.62, *p* < 0.01), had a significant influence in the model. Pairwise comparisons revealed that for strong brands, evaluation differed between the monobrand and the co-brand (*M_mono_before_* = 7.13, *SD* = 0.07 vs. *M_co-brand_* = 6.67, *SD* = 0.07, *p* < 0.01) (see Figure 4). Fit significantly moderated this time effect, as revealed by model 2, estimated in MEMORE [44]. For each unit increase in fit, there is a 0.25 decrease in the difference between the previous evaluation of the monobrand and the co-brand (−0.25, CI [−0.31, −0.19]). Hence, the difference between the previous evaluation of the monobrand and the co-brand increased at low levels of fit (*β_low_fit_* = 1.01, CI [0.82, 1.21]) and at moderate levels of fit (*β_moderate_fit_* = 0.45, CI [0.31, 0.59]), while at high fit levels, the difference was not pronounced (*β_high_fit_* = −0.11, CI [−0.30, 0.09]). This result corroborates H2. 

In contrast, for the brands of moderate strength, no significant difference was observed between the monobrand and the co-brand (*M_mono_before_* = 6.05, *SD* = 0.07 vs. *M_co-brand_* = 5.96, *SD* = 0.07, *p* = 0.36) (see Figure 4). This result points to the moderating effect of brand strength and confirms H3: co-brands constituting strong brands are evaluated as being worse than the monobrands, while this effect is not observed for moderate monobrands and co-brands. 

For the strong brands, no significant difference was observed between the monobrand’s evaluation before and after exposure to the co-brand (*M_mono_before_* = 7.13, *SD* = 0.07 vs. *M_mono_after_* = 7.17, *SD* = 0.07, *p* = 1) (see Figure 4). This finding validates the results from study 1 and leads to the rejection of H4. Results from another model 2, estimated in MEMORE, revealed the significant moderating effect of fit (−0.06, CI [−0.11, −0.01]), with this effect only being significant at high fit levels (*β_high_fit_* = −0.17, CI [−0.34, −0.01]). 

**Figure 5:**
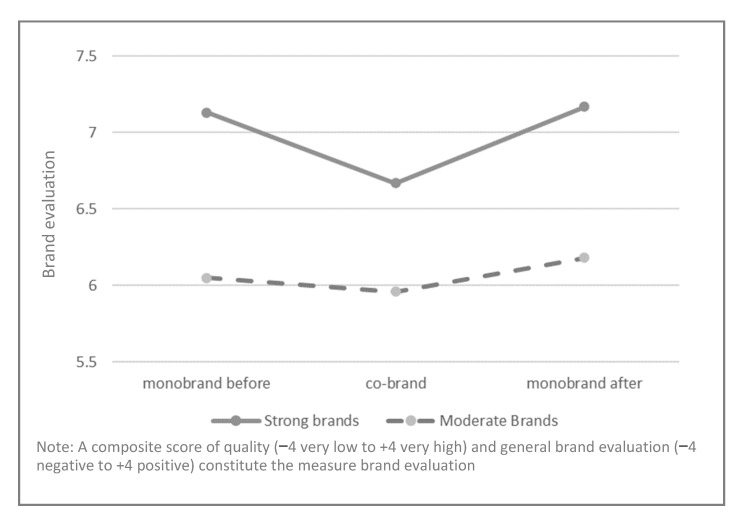
Mean comparisons of study 2.

In a similar fashion to the strong brands, the moderating role of fit as postulated in H2 is confirmed; fit significantly moderated the effect of time on brand evaluation (−0.25, CI [−0.31, −0.19]) for the moderate brands. Model 2 (MEMORE) revealed that the difference between the average evaluation of the monobrands before exposure to the co-brand (phase 1) and the co-brand evaluation (phase 2) is larger at low fit levels (*β_low_fit_* = 0.66, CI [0.46, 0.85], while no significant moderating effect was observed at moderate fit levels (*β_moderate_fit_* = 0.09, CI [−0.04, 0.23]), and the difference is reduced at high fit levels (*β_high_fit_* = −0.47, CI [−0.66, −0.27]). Replicating the pattern observed for the strong brands, the monobrand evaluation did not differ between phase 1 and phase 3 (*M_mono_before_* = 6.05, *SD* = 0.07 vs. *M_mono_after_* = 6.18, *SD* = 0.07, *p* = 0.12) (see Figure 5). The estimation of model 2 (MEMORE) with the two measurement points, moderate monobrand before and moderate mono-brand after, revealed that fit between the two monobrands significantly impacted by the effect of time (−0.08, CI [−0.12, −0.04]) at moderate (*β_moderate_fit_* = −0.12, CI [−0.21, −0.04]) and high fit levels (*β_high_fit_* = −0.31, CI [−0.43, −0.19]). This result confirms H2.

## 3. Discussion

The current research addresses three gaps in the literature: (1) potential negative impacts of a co-branding strategy on the co-brand’s and the monobrands’ evaluations, (2) the moderating effects of brand strength and brand fit, and (3) adaptive learning models as an explanation for an overexpectation effect in the context of cobranding. 

On a quite general level, our findings confirm the relevance of brands as an extrinsic cue for brand evaluation. Across two experiments with data representative of the population under investigation, we investigated how co-branding as a common strategy in food branding impacts brand evaluations. We relied on real-world brands from six different food product categories to test the major claim that co-branding strategies do not always benefit food products. 

Our most notable contribution to extant research concerns the absence of any effect or even no effect of a co-branding strategy consisting of two strong food brands on brand evaluations. This result validates prior research that did not observe any effect or found a negative effect, of co-branding strategies on brand equity [12,26]. However, in contrast to extant studies, the focus of our research was the potential negative effect of co-branding strategies. Hence, we advance the extant literature by explaining the decrease in brand evaluations on a co-brand level through adaptive learning models [18,21]. Adaptive learning models suggest that different cues (e.g., brand names) compete for an outcome and that the strength between one cue and an outcome (e.g., brand evaluation) is updated based on new information (e.g., a co-branding arrangement) [15].

More specifically, drawing on these models, we show that the combination of two strong brands results in an overexpectation effect [18]. This effect implies that consumers’ expectations are too high to be met by the co-branded product, resulting in a decrease in evaluations for each monobrand. As a result, consumers evaluate the co-brand as being worse than the average of the two monobrands. This finding confirmed H1. In general, the effects are stable among all the experiments using different stimuli and different experimental procedures. 

Surprisingly, we did not observe a negative feedback effect on the monobrand after exposure to the co-brand. In other words, the monobrand’s evaluation was not different after being shown the co-brand when compared to the evaluation given before the exposure. Accordingly, it can be concluded that despite the lower brand evaluations on a co-brand level, this negative effect does not spillover to monobrands. This finding was unexpected, leading to the rejection of H4, and contradicts previous research reporting a negative effect of co-branding on the partnering monobrands [15,32,33,34]. However, it must be noted that the co-branding strategy did not benefit the monobrand. Hence, overall, the co-branding strategy did not result in the expected increase of brand evaluations on a co-brand level and did not affect brand evaluations on a monobrand level. 

In addition to the main effect of co-branding on brand evaluations, we further identified two conditions that attenuate this negative effect. First, by drawing on previous literature [17,29,30,31], we suggested and validated the moderating effect of brand fit. While brand fit has been acknowledged to determine to a certain extent co-branding success, no study thus far has explored how brand fit diminishes the overexpectation effect. In adding to the literature, we observed that low and moderate levels of fit increase the negative effect of co-branding on brand evaluations, while high fit levels did not moderate the difference between the monobrand evaluation and the co-brand evaluation (H3). This finding emphasizes the relevance of a good fit between the partnering brands in a co-branding arrangement. Brand managers should pay particular attention to the complementarity of brands when pursuing a co-branding strategy so that the brand attributes of the partnering brand complement each other.

In addition, we identified brand strength as an important condition for the overexpectation effect in a co-branding context (H3). While we replicated study’s 1 results for the overexpectation effect for strong brands, we did not find an overexpectation effect for moderate brands. No decrease in brand evaluations occurred for moderate co-brands when compared to the monobrands was observed. This result further strengthened our reasoning that only co-brands consisting of strong brands are evaluated as being worse than the average of the two monobrands, something caused by the overexpectation effect. Although not statistically significant, the co-brand of moderate monobrands was also evaluated as being lower than the preceding view of the monobrands. This finding is of high scientific and practical relevance. From a scientific perspective, the presence of the overexpectation effect for strong brands only offers further empirical evidence on the applicability of adaptive learning models in the context of co-branding. Following our theoretical reasoning, we found that the high expectations associated with strong brands result in a decrease in brand evaluations to reduce the discrepancy between the expected and the actual outcome. From a practical viewpoint, this result suggests that brand managers should be cautious in pursuing co-branding strategies involving two strong brands. On the contrary, brands of moderate strength are less likely to cause overexpectation, and hence can be considered for co-branding strategies. 

Despite the robustness of our findings, the designs used here have some limitations which offer avenues for future studies. We only tested the effect of co-branding on brand quality and brand evaluation, which were summarized as one overall brand evaluation score for the analysis. However, companies that pursue co-branding strategies might pursue other aims as well. For instance, if manufacturers of new brands strive to increase brand awareness, co-branding might indeed be a promising strategy. Future studies might replicate our findings with alternative dependent variables, for instance, willingness to pay. As another alternative outcome variable, consumers’ brand choice could be studied in a choice experiment (either in a laboratory or a real-world setting). Although both studies relied on representative samples, the samples were only representative of the country under investigation. Hence, future studies might validate the findings with samples from other cultures. Finally, the reliance on self-reported scales has some limitations, which might be overcome by relying on alternative measurement methods, such as an implicit association test, or even protocols to gain deeper insights into the brand associative learning process of consumers.

## Figures and Tables

**Table 1:** Stimuli in Experiment 1 and Experiment 2.

Product Category	Strong Brand	Popularity 2021	Moderate Brand	Popularity 2021
Pastry	Ölz	85%	Bäckerland	nA
Juices	Rauch	72%	MeinObst	nA
Mineral water	Vöslauer	84%	Rogaska	nA
Beer	Gösser	85%	Zillertal	22%
Chocolate	Milka	90%	Schogetten	64%
Soft drinks	Almdudler	92%	Radlberger	62%

Notes: nA indicates that the respective brand was not mentioned in the popularity ranking of the respective product category, Sources: [36,37,38,39,40,41].

## Data Availability

For each experiment, the data sets are accessible online: https://data.mendeley.com/datasets/t876rkzyf4/2 (accessed on 10 July 2022).
